# Circulating microparticles carry oxidation-specific epitopes and are recognized by natural IgM antibodies^1^[S]

**DOI:** 10.1194/jlr.P054569

**Published:** 2015-02

**Authors:** Dimitrios Tsiantoulas, Thomas Perkmann, Taras Afonyushkin, Andreas Mangold, Thomas A. Prohaska, Nikolina Papac-Milicevic, Vincent Millischer, Caroline Bartel, Sohvi Hörkkö, Chantal M. Boulanger, Sotirios Tsimikas, Michael B. Fischer, Joseph L. Witztum, Irene M. Lang, Christoph J. Binder

**Affiliations:** * Department of Laboratory Medicine, Medical University of Vienna, Vienna, Austria; ** Department of Cardiology, Medical University of Vienna, Vienna, Austria; ††† Department of Blood Group Serology Transfusion Medicine, Medical University of Vienna, Vienna, Austria; † Center for Molecular Medicine (CeMM) of the Austrian Academy of Sciences, Vienna, Austria; § Christian Doppler Laboratory for Innovative Therapy Approaches in Sepsis, Krems, Austria; †† Medical Research Center and Department of Medical Microbiology and Immunology, Institute of Diagnostics, University of Oulu, Oulu, Finland; §§ INSERM U970, Paris Cardiovascular Research Center - PARCC, Paris, France; *** Department of Medicine, University of California San Diego, La Jolla, CA

**Keywords:** immunoglobulin M antibodies, malondialdehyde, acute coronary syndrome

## Abstract

Oxidation-specific epitopes (OSEs) present on apoptotic cells and oxidized low density lipoprotein (OxLDL) represent danger-associated molecular patterns that are recognized by different arcs of innate immunity, including natural IgM antibodies. Here, we investigated whether circulating microparticles (MPs), which are small membrane vesicles released by apoptotic or activated cells, are physiological carriers of OSEs. OSEs on circulating MPs isolated from healthy donors and patients with ST-segment elevation myocardial infarction (STE-MI) were characterized by flow cytometry using a panel of OSE-specific monoclonal antibodies. We found that a subset of MPs carry OSEs on their surface, predominantly malondialdehyde (MDA) epitopes. Consistent with this, a majority of IgM antibodies bound on the surface of circulating MPs were found to have specificity for MDA-modified LDL. Moreover, we show that MPs can stimulate THP-1 (human acute monocytic leukemia cell line) and human primary monocytes to produce interleukin 8, which can be inhibited by a monoclonal IgM with specificity for MDA epitopes. Finally, we show that MDA^+^ MPs are elevated at the culprit lesion site of patients with STE-MI. Our results identify a subset of OSE^+^ MPs that are bound by OxLDL-specific IgM. These findings demonstrate a novel mechanism by which anti-OxLDL IgM antibodies could mediate protective functions in CVD.

Inflammation and immunity are recognized as important modulators of CVDs. A large body of evidence supports a role for oxidized LDL (OxLDL) as mediator of the inflammatory responses that propagates atherosclerotic lesion formation (1). IgGs to OxLDL are present in lesions, and their titers in plasma have been found to be positively (though inconsistently) associated with CVD risk (2). On the other hand, increasing epidemiological evidence demonstrates that high levels of IgM antibodies to OxLDL are inversely associated with CVD, suggesting a protective role for these antibodies (3–7). Moreover, a number of experimental studies in mouse models of atherosclerosis support a protective role for IgM (8–10). Most anti-OxLDL IgMs are believed to be so-called natural antibodies (NAbs), which are preexisting antibodies with germ-line or near germ-line encoded variable regions (2). However, it is not known how anti-OxLDL IgM NAbs confer protection and if OxLDL itself represents the actual/only target antigen of such antibodies in vivo.

Anti-OxLDL NAbs have specificity for various oxidation-specific epitopes (OSEs). OSEs are generated by the modification of proteins and lipids with reactive breakdown products resulting from lipid peroxidation. Prominent examples include oxidized glycerophospholipids (OxGPs) or malondialdehyde (MDA) and 4-hydroxynonenal. We have previously shown that up to 30% of IgM NAbs have specificity for OSEs (11). Importantly, the same OSEs found on OxLDL have also been shown to be present in various inflammatory tissues (11–13) and on the surface of apoptotic cells (11, 14, 15). Thereby, they tag “metabolic waste” marking it for removal by the immune system. OSEs have also been shown to trigger inflammatory responses in macrophages and endothelial cells [ECs; e.g., the secretion of interleukin (IL) 8] (15–17).

Microparticles (MPs) are defined as small anucleoid phospholipid vesicles (0.1–1 μm) that are released from activated or dying cells including platelets, red blood cells (RBCs), ECs, and monocytes and are present in the circulation (18). MPs are formed when membrane phospholipid asymmetry is lost resulting in phosphatidylserine (PS) exposure (19). Elevated levels of circulating MPs have been shown to be associated with CVD (18–20). For example, plasma MPs are increased in patients with acute coronary syndrome (ACS) (21) and individuals with high atherothrombotic risk, as well as in patients with venous thromboembolism (20). Several studies found proinflammatory as well as prothrombotic activities for MPs. PS exposure on MPs enhances coagulation by promoting assembly of clotting factors (19). Moreover, leukocyte-derived MPs in particular can display procoagulatory tissue factor (19). In addition, MPs can activate cells through cell surface receptors via the ligand molecules they carry on their surface, or they can transfer biological material (e.g., RNA and proteins) to recipient cells, thereby modulating their function (22). For example, MPs isolated from human atherosclerotic plaques were shown to mediate monocyte adhesion on ECs by directly transferring intracellular adhesion molecule-1 (ICAM-1) (23). Monocyte-derived MPs were found to transfer IL-1β and other components of the inflammasome to ECs, resulting in the expression of ICAM-1, vascular cell adhesion molecule-1, and E-selectin (24). Nevertheless, the proinflammatory moieties mediating effects of MPs are far from fully characterized. For example, also bioactive lipid moieties have been suggested to be involved (25).

We have previously demonstrated that apoptotic cells are bound by OSE-specific IgM NAbs, which neutralize their proinflammatory properties in vitro and promote their clearance in vivo (11, 12). In this study, we investigated whether circulating MPs isolated from healthy individuals and patients with acute myocardial infarction (AMI) carry OSEs. We report the identification of a novel subset of proinflammatory circulating MPs in both healthy subjects and patients that is characterized by the presence of OSEs, most prominently MDA modifications. Many of these MPs also carry OSE-specific neutralizing IgM, suggesting a novel way by which OSE-specific IgM may protect from CVD.

## MATERIALS AND METHODS

### Collection of blood samples and isolation of circulating MPs

Blood samples were obtained from the antecubital vein of healthy individuals of two independent cohorts: *a*) 18 healthy individuals (10 female, 8 male) with a mean age of 28.4 and 28.7 years, and *b*) 15 healthy individuals, 10 male and 5 female, with mean age 32.1 and 32.8 years, respectively. Moreover, blood samples were collected from a 6 French femoral sheath (herein after referred to as periphery) and from the coronary aspiration catheter of 14 patients during primary percutaneous intervention (pPCI) for ST-segment elevation myocardial infarction (STE-MI), as described (26). Detailed patient characteristics are shown in supplementary Table 1. Patients received 250 mg aspirin and were treated with unfractionated heparin to achieve an activated clotting time ≥300 s. A total of 10 ml blood was aspirated with a Pronto® V3 thrombus extraction catheter or the Medtronic Export Aspiration Catheter at the site of thrombotic occlusion prior to any intervention. Because of a 2 ml 0.9% sodium chloride flush of the aspiration catheter prior to thrombectomy, all analyses were normalized for hematocrite.

Blood samples were collected into K2EDTA-containing collection tubes (Vacutainer® tubes, Becton Dickinson). Samples were immediately centrifuged at room temperature for 30 min at 2,000 *g* to obtain platelet-poor plasma. In some experiments, plasma was additionally treated with 40 µmol of butylated hydroxytoluene (BHT). The resulting MP-containing plasma was carefully removed without disturbing the cell pellet. An aliquot of the plasma sample was stored at −20°C, and the rest was transferred to autoclaved tubes and centrifuged at 21,000 *g* for 30 min at 4°C to pellet MPs. In some experiments, plasma was centrifuged for 2 min at 13,000 *g* to remove remnant platelets and aggregates prior to the ultracentrifugation step. After centrifugation, plasma was harvested and pelleted MPs were resuspended and washed with Dulbecco’s PBS (DPBS; Sigma Aldrich) at 18,000 for 30 min. This washing step was repeated at least three times. Finally, MPs were resuspended in DPBS and stored at −20°C and/or used for further experiments. All studies were conducted after patients’ or healthy individuals’ written informed consent under the approval of the Ethics Committee of the Medical University of Vienna (EK-N: 303/2005, 2177/2013, and 2051/2013).

### Inclusion criteria of STE-MI patients

Patients in the setting of STE-MI were included if all of the following criteria applied: *1*) chest pain at the time of coronary angiography associated with ST-segment elevation in two or more contiguous leads, *2*) Thrombolysis In Myocardial Infarction 0–1 flow in a native culprit coronary artery and an intraluminal filling defect suggestive of thrombus within 50 mm of the respective coronary ostium, *3*) absence of complete heart block, and *4*) no thrombolytic therapy/or treatment with glycoprotein IIbIIIa antagonists.

### Flow cytometric analysis of MPs

To assess the presence of PS, isolated MPs were stained with annexin V conjugated to phycoerythrin (PE) (eBiosciences) in calcium-containing buffer (eBiosciences). To determine the presence of OSEs on their surface, isolated MPs were stained with 2 µg/ml of biotinylated or 40 µg/ml of unconjugated antibodies in filtered (0.1 µm) PBS containing 0.5% fatty acid free BSA (PAA Laboratories) of the following IgM primary antibodies: anti-keyhole limpet hemocyanin (MM-30; Becton Dickinson) as isotype control; T15/E06 specific for oxidized phosphocholine-containing phospholipids; and NA-17, E014, and LR04, which are directed against MDA, including advanced malondialdehyde-acetaldehyde (MAA) adducts (11, 27–29). The samples were incubated with the antibodies for 30 min at 4°C. Then MPs were pelleted at 18,000 *g* for 15 min. Supernatants were removed, and avidin conjugated to FITC (Becton Dickinson) or anti-mouse IgM conjugated to allophycocyanin (APC) (II/41; eBiosciences) was added at a final concentration of 0.5 µg/ml or 1 µg/ml, respectively, and incubated for 20 min in darkness at 4°C. To identify MPs that carry both MDA and phosphocholine epitopes, MPs were sequentially stained with T15/E06, then anti-mouse IgM FITC at 2.5 µg/ml (II/41; Becton Dickinson), followed by LR04 and then anti-mouse IgM APC. To determine surface-bound human IgM, MPs were stained with an anti-human IgM-PE (MHM 88; Becton Dickinson) or with the corresponding isotype control antibody (MOPC-21; Becton Dickinson) and incubated for 15 min in darkness at 4°C.

To identify the cellular origin, MPs were stained with anti-human CD235a conjugated to R-PE (HIR 2), anti-human CD41a-FITC (HIP 8), anti-human CD14-PercP Cy5.5 (61D3), or biotinylated anti-human CD31 (WM59), all bought from eBiosciences, and incubated for 30 min in darkness at 4°C. After incubation with antibodies, MPs were pelleted at 18,000 *g* for 15 min, and the samples were then incubated with 0.5 µg/ml streptavidin-PercP (Biolegend) for 20 min in darkness at 4°C (Becton Dickinson).

To quantify circulating MPs in the peripheral and coronary blood of AMI patients, diluted plasma samples were stained with annexin V-PE. All samples were acquired on a FACS Calibur (Becton Dickinson) for 30 s at low speed. Forward and sideward scatter were set at logarithmic gain. MPs were identified as annexin V-positive events with size ≤1 µm, using monodisperse polystyrene MP size standards with a mean size of 1 µm as reference. MP-free buffer was acquired in order to exclude false events due to noise (supplementary Fig. 1). Samples were analyzed on a FACS Calibur (Becton Dickinson). All acquired data were analyzed with Flow Jo software (Treestar).

### In vitro generation of platelet-derived MPs

Purified platelets were obtained by apheresis from the Department of Transfusion Medicine of the Medical University of Vienna and incubated with 10 µM of ionomycin in PBS containing 1 mM CaCl_2,_ for 30 min at 37°C. After adding 5 mM EDTA to stop the reaction, cells and debris were pelleted by sequential centrifugation at 2,000 *g* and 3,900 *g* for 10 min, respectively. After centrifugation, the MP-containing supernatant was carefully removed, and MPs were pelleted and washed with DPBS and centrifuged at 18,000 *g *for 30 min. This washing step was repeated at least three times. Finally, MPs were resuspended in DPBS + 0.01% BHT and stored at −20°C for further experiments. The MP total protein amount was estimated using the BCA protein assay (Pierce).

### Flow cytometric analysis of platelets

Purified platelets were stained with 10 µg/ml of LR04 or an isotype control antibody (MM-30; Biolegend) for 30 min at 4°C. After washing in filtered PBS + 0.5% BSA, cells were pelleted at 500 *g* and incubated with 0.25 µg/ml of anti-IgM APC (II/41; eBiosciences) for 20 min in darkness at 4°C. Cells were washed again and samples were acquired on a FACS Calibur (Becton Dickinson), and data were analyzed using Flow Jo software (Treestar).

### Elution of MP-associated IgM

Isolated and extensively washed circulating MPs were lysed in RIPA buffer containing 50 mM Tris-HCl, 150 mM NaCl, 1% NP-40, 1% sodium deoxycholate, and 0.1% SDS (pH = 7.6) supplemented with a complete protease inhibitor cocktail (Roche Applied Science). After overnight incubation at 4°C, the MP lysates were transferred to microdialysis cups with an molecular weight cut-off of 10 kDa (Micro DispoDialyzers, Harvard Apparatus) and extensively dialyzed against PBS containing 0.27 mM EDTA.

### Antigen binding of monoclonal IgM antibodies

The specificity of T15/E06, LRO4, NA17, and E014 is extensively documented in the literature (11, 27–29). To confirm these specificities with the aliquots used, we performed direct binding studies to native BSA, PC-BSA, or MDA-BSA (which contains different types of MDA adducts) using chemiluminescent ELISA as described (11).

### Antibody measurements and competition assay

Total IgM and IgM antibodies to native LDL, Cu^2+^-oxidized LDL (Cu-OxLDL), and MDA-modified LDL (MDA-LDL) were measured as described previously (11). In brief, 5 µg/ml of native LDL, Cu-OxLDL, and MDA-LDL diluted in PBS containing 0.27 mM EDTA and 0.02% sodium azide (PBS/EDTA) were coated to 96-well white round-bottomed MicroFluor microtiter plates (ThermoLabsystems) and incubated overnight at 4°C. Coated plates were then blocked with TBS containing 1% BSA (TBS/BSA) for 1 h at room temperature. After washing three times with PBS/EDTA, 50 µl of diluted plasma (1:10,000) or MP lysates (1:20) in TBS/BSA were added and incubated overnight at 4°C. Plates were washed again three times with PBS/EDTA and binding of the IgM antibodies was detected with an alkaline phosphatase-conjugated goat anti-human IgM (A3437; Sigma Aldrich) diluted 1:30,000 in TBS/BSA. Following an incubation time of 1 h at room temperature, the wells were washed three times as described above, followed by a single rinse with distilled water. Then, 25 µl of a 30% LumiPhos Plus solution in distilled water (Lumigen Inc.) was added. After 90 min, the light emissions were measured on a WALLAC VIKTOR II luminometer (Perkin Elmer) and expressed as relative light units per 100 ms. For the determination of total IgM, plates were coated with 5 µg/ml of the polyclonal goat anti-human IgM antibody (I2386; Sigma Aldrich), and IgM in plasma (1:10,000) or MP eluates (1:20) diluted in TBS/BSA were measured as above. The ratio of antigen-specific IgM per total IgM was calculated for each sample.

To quantify the IgM antibodies against MDA-LDL in the peripheral and coronary blood of the AMI patients, plasma was diluted (1:800) in TBS/BSA, and binding to coated MDA-LDL was measured as described. Obtained data were normalized to hematocrit values in the periphery and coronary blood, respectively.

To determine the specificity of the MP-bound IgM, an ELISA-based immunocompetition assay using native LDL, Cu-OxLDL, or MDA-LDL as competitors was performed. MP lysates (1:50) were diluted in TBS/BSA and mixed 1:1 with 50 µg/ml of native LDL, Cu-OxLDL, or MDA-LDL, and binding to coated MDA-LDL was measured as described above.

### Isolation of human primary monocytes

Blood was obtained from the antecubital vein of healthy individuals (n = 6) as described above. Peripheral monocytes were isolated using Ficoll-Paque (Sigma) followed by positive selection using CD14 Microbeads (Miltenyi) according to the manufacturer’s instructions. The purity of the different preparations was evaluated by flow cytometry using anti-human CD14-PercP Cy5.5 (61D3). Samples were analyzed with a FACS Calibur (Becton Dickinson), and data were analyzed using the Flow Jo software (Treestar). The purity of all preparations was found to be >96%.

### In vitro stimulation and neutralization assay

To characterize the proinflammatory ability of MPs in vitro, 5 × 10^4^ THP-1 or primary human monocytes (after resting for 36 h in RPMI + 10% FBS) were stimulated with 200 µg/ml of in vitro generated platelet-derived MPs for 8 h at 37°C in RPMI media containing 50 µg/ml BSA. In parallel experiments, MPs were cocultured with 25 or 50 µg/ml of either LR04 or control antibody (MM-30; Biolegend). At the end of the 8 h stimulation, cell-free supernatants were collected by pelleting the cells in a 96-well V-bottom plate at 400 *g* for 3 min. IL-8 protein levels were quantified in the supernatant by ELISA using a commercial kit (Becton Dickinson).

### Statistical analyses

Statistical analyses were performed using Graph Pad Prism 5 for Windows (Graph Pad Software). Two data groups were compared using Student’s unpaired or paired *t*-test. To analyze multiple group data, either one-way or two-way ANOVA tests followed by Bonferroni’s multiple comparison tests were performed as appropriate. For correlation analysis, the Pearson’s test was used. Data are presented as mean ± SEM. A *P* value of <0.05 was considered significant.

## RESULTS

### A subset of circulating MPs carry OSEs

We and others have previously shown that apoptosis of ECs, thymocytes, and Jurkat T cells induced by various triggers, such as serum deprivation, staurosporine, dexamethasone, PMA, and UV irradiation, results in the formation of OSEs on the surface of these cells (11, 14, 15, 29, 30). Moreover, in vitro Fe^2+^ and tert-butyl hydroperoxide-treated microvesicles of ECs as well as microvesicles of THP-1 cells treated with unesterified cholesterol have been found to carry OSEs (31, 32). To test whether OSEs are indeed present on MPs in vivo, we isolated MPs from plasma of healthy individuals by sequential centrifugation (supplementary Fig. 1), and the presence of OSEs on the surface of annexin V^+^ MPs was determined by flow cytometry using previously characterized monoclonal anti-OxLDL IgM NAbs with specificity for PC (T15/E06) and MDA-type epitopes (LR04, NA17, and E014), respectively (11, 27–29). These NAbs bind OxLDL and exhibit a selective specificity for certain OSEs (11, 27–29) (supplementary Fig. 2).

**Fig. 1.**
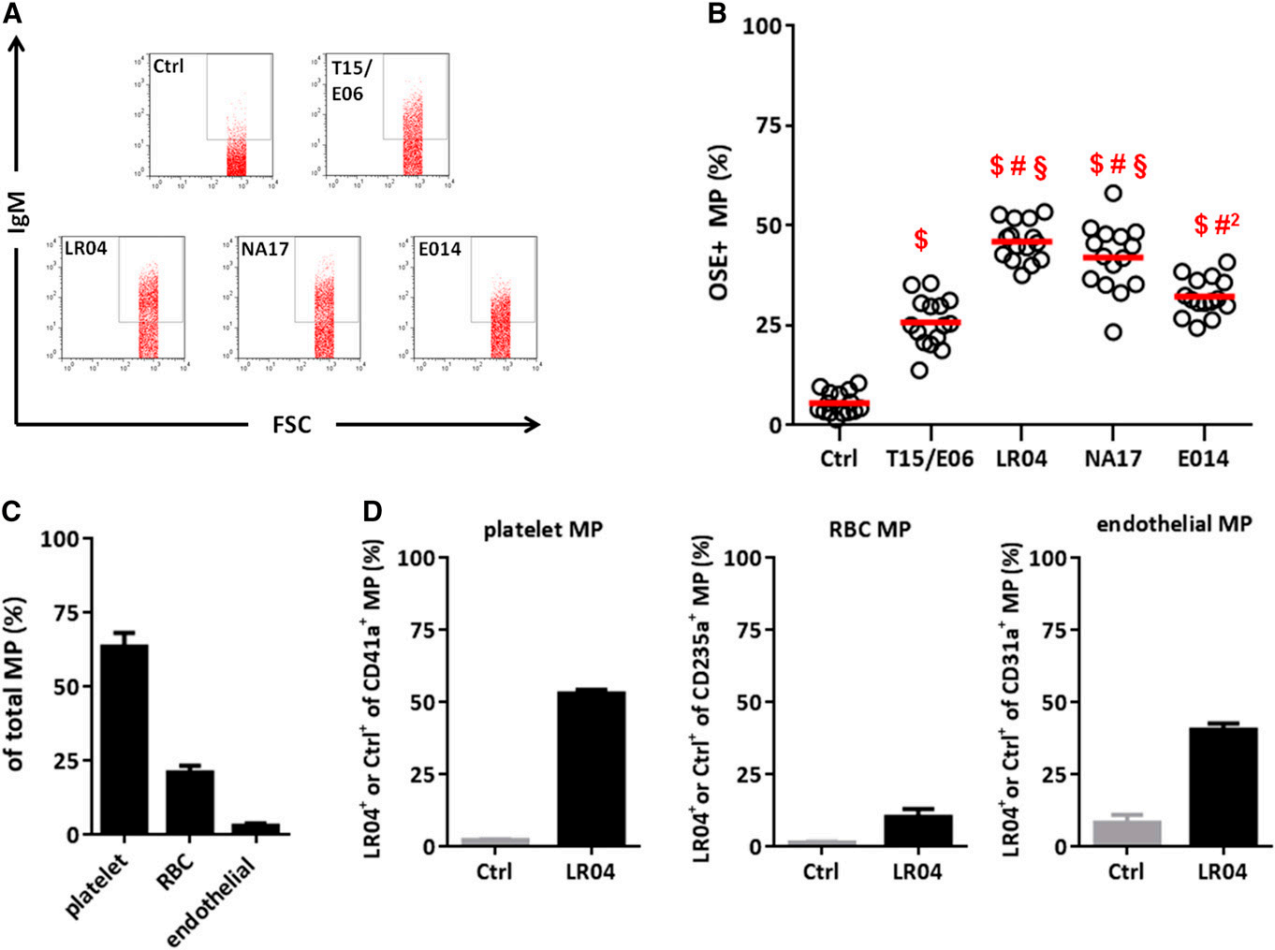
A subset of circulating MPs carry OSEs. A, B: MPs isolated from plasma of healthy volunteers were stained with annexin V and OSE-specific IgM NAbs including T15/E06 (specific for PC), LR04, NA17, and E014 (specific for MDA-type epitopes) and analyzed by flow cytometry. A: Representative flow cytometry plots of stained MPs. B: Quantification of the percentages of MPs with positive staining for each antibody. Bonferroni’s multiple comparison test; ^$^ *P* < 0.0001 compared with isotype control, ^#^ *P* < 0.0001 and ^#2^ *P* < 0.05 compared with T15/E06, and ^§^ *P* < 0.001 compared with E014. Black circles depict MPs from individual donors identified by annexin V^+^ and size ≤1 µm. C, D: MPs were stained with anti-CD41a (platelets), anti-CD235a (RBCs), and anti-CD31 (ECs) to identify their cellular origin and with LR04 to assess MDA**^+^** MPs within each cellular fraction. Distribution of cellular origin (C) and percentages of LR04^+^ MPs (D) of MPs of all donors. Data represent mean ± SEM.

A subset of circulating MPs of all tested individuals were bound by OSE-specific IgMs, while an isotype control antibody showed only minimal binding (**Fig. 1A**). On average, 26 ± 2% of MPs were bound by the OxGP-specific NAb T15/E06 (Fig. 1B). Strikingly, 46 ± 1% and 42 ± 2% of MPs showed reactivity with the MDA-specific antibodies LR04 and NA17, respectively (11, 27). In addition, the MDA-specific antibody E014 bound 32 ± 1% of MPs, suggesting specificity for a different type of MDA adduct. A similar percentage of OSE-carrying MPs was found in the plasma of a second cohort of healthy individuals (supplementary Fig. 3). Moreover, costaining of circulating MPs with LR04 and T15/E06 antibodies revealed that PC epitopes are nearly exclusively present on MDA-carrying MPs (supplementary Fig. 4). To rule out that the presence of OSEs was due to ex vivo oxidation, BHT was added to the plasma, in addition to EDTA, before isolating MPs, as addition of BHT to plasma has been shown to prevent ex vivo oxidation of LDL (33). Supplementation of plasma with BHT did not affect the presence of OSEs (supplementary Fig. 5).

### MDA epitopes are present on circulating MPs irrespective of their cellular origin

As circulating MPs originate from different parental cells, we investigated the cellular origin of the MP preparation, which contained 85 ± 1% annexin V^+^ particles. MP origin was analyzed using antibodies specific for CD41a (platelets), CD235a (RBCs), and CD31 (ECs). Consistent with previous reports (20), a majority (64 ± 5%) of MPs were of platelet origin, while 21 ± 2% were RBC-derived and 3 ± 1% were derived from ECs (Fig. 1C). MDA-carrying MPs were present irrespective of the cellular origin of MPs (Fig. 1D). To further corroborate our results and to test whether MDA epitopes can also be formed in vitro during the process of MP formation, we generated MPs from platelets by Ca^2+^ ionophore stimulation. While the MDA-specific antibody LR04 did not bind to the surface of platelets, a large part of MPs generated from these cells showed strong binding (supplementary Fig. 6).

### Circulating MPs carry surface bound IgM with specificity for MDA-LDL

**Fig. 2.**
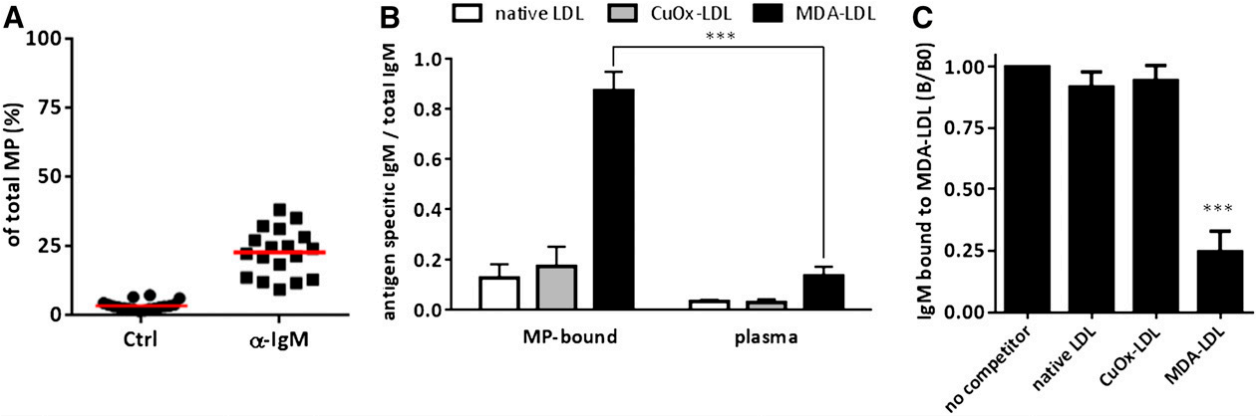
Circulating MPs carry IgMs that have specificity for MDA-LDL. A: IgM antibodies are bound on the surface of circulating MPs. MPs isolated from healthy volunteers were stained with anti-human IgM and an isotype control antibody and analyzed by flow cytometry. Symbols depict percentages of MPs with bound IgM of individual donors. B: IgM antibodies eluted from circulating MPs are enriched for IgM with specificity for MDA-LDL compared with IgM in plasma. Binding of MP-eluted and plasma IgM to native LDL, Cu-OxLDL, and MDA-LDL was measured by ELISA, and the ratio of antigen-specific IgM/total IgM was calculated. Shown are the mean ± SEM results of four samples. *** *P* < 0.0001; Bonferroni’s multiple comparison test. C: Competition immunoassay. Binding of MP-eluted IgM to coated MDA-LDL in the presence of soluble LDL, Cu-OxLDL, and MDA-LDL. Data are expressed as a ratio of the binding in the presence of competitor divided by the binding in the absence of competitor (*B/B_0_*). Shown are mean ± SEM data of four donors. *** *P* < 0.0001; Bonferroni’s multiple comparison test.

Because OSEs are a dominant target of IgM NAbs (11), we tested whether part of the isolated MPs were already bound by IgM. Indeed, we found that 23 ± 2% of circulating MPs had endogenous IgM bound on their surface even after extensive washing (**Fig. 2A**). Next, we eluted IgM from lysed MPs and assessed the binding to MDA-LDL and Cu-OxLDL, two model antigens for OSEs, in comparison to circulating IgM in plasma of the same individuals. Normalization of antigen-specific IgM to total IgM revealed that IgM antibodies to MDA-LDL represented >80% of total IgM in MP eluates, while plasma IgM to MDA-LDL was ∼15% of total IgM (Fig. 2B). The latter findings are consistent with our previous demonstration that MDA-LDL-specific IgMs represent ∼15% of IgM NAbs in mice (11). The specificity of the MP-eluted IgM for MDA-LDL was tested in immunocompetition assays, in which neither soluble native LDL nor Cu-OxLDL competed for the binding to coated MDA-LDL, while soluble MDA-LDL inhibited the binding by >75% (Fig. 2C). Thus, MP-associated IgMs are primarily composed of IgM with specificity for MDA-LDL. These data are consistent with the presence of OSEs on a subset of circulating MPs and support the notion that MDA adducts are dominant.

### MDA adducts contribute to the proinflammatory effect of MPs

**Fig. 3.**
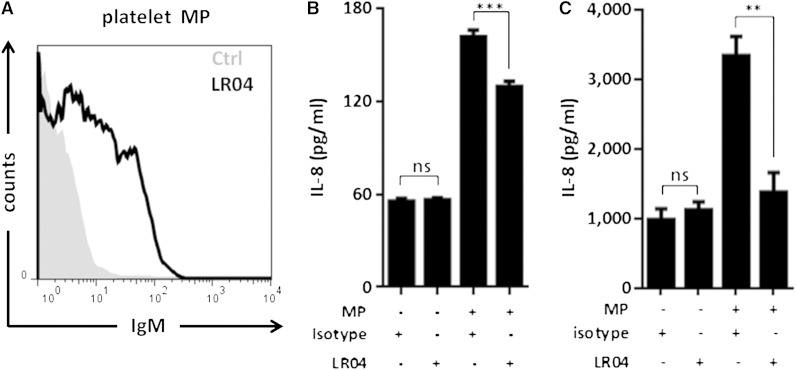
LR04, an MDA-specific IgM NAb, decreases the proinflammatory effect of platelet MPs. A: Platelet-derived MPs stained with the MDA/MAA-specific LR04 or isotype antibody and analyzed by flow cytometry. B: Stimulation of THP-1 human monocytes for 8 h with in vitro generated platelet-derived MPs resulted in IL-8 secretion, which was inhibited when MPs were preincubated with LR04 compared with isotype control. Data are from one experiment representative of four in triplicate determinations. C: Stimulation of primary human monocytes isolated from healthy donors (n = 6) for 8 h with in vitro generated platelet-derived MPs resulted in IL-8 secretion, which was inhibited when MPs were preincubated with LR04 but not an isotype control. Data are from two independent experiments. Data are presented as mean ± SEM (ns, not significant; ** *P* < 0.01, *** *P* < 0.001; Bonferroni’s multiple comparison test or paired *t*-test).

We and others have previously shown that MAA-modified BSA induces proinflammatory responses in various cells, including IL-8 secretion by THP-1 cells (16, 34). To test whether MPs induce chemokine expression via MDA epitopes, we determined IL-8 secretion by THP-1 cells or primary human monocytes that were stimulated with platelet-derived MPs in the presence of the MDA/MAA-specific IgM LR04 or an isotype control. A subset of platelet-derived MPs were bound by LR04, but not an isotype control (**Fig. 3A**). MP stimulation induced secretion of IL-8 by both THP-1 and primary human monocytes. Coincubation with the MDA-specific antibody LR04, but not an isotype control, inhibited MP-induced IL-8 production by >25% and >90% in THP-1 cells and primary monocytes, respectively (Fig. 3B, C). The more robust inhibition found in primary monocytes may be explained by previously observed differences between THP-1 cells and primary monocytes in their functional responses and suggests that primary monocytes are more sensitive to MDA-dependent effects (35). In contrast, LR04 did not inhibit the IL-8 secretion induced upon stimulation with the specific Toll-like receptor 4 ligand Di[3-deoxy-D-manno-octulosonyl]-lipid A (data not shown). Collectively, these data demonstrate the capacity of MDA-specific natural IgM antibodies to inhibit inflammatory effects of MPs and indicate that the proinflammatory properties of MPs are to a significant extent mediated by MDA/MAA-epitopes.

### MDA-carrying MPs are increased at the site of the coronary occlusion in STE-MI

**Fig. 4.**
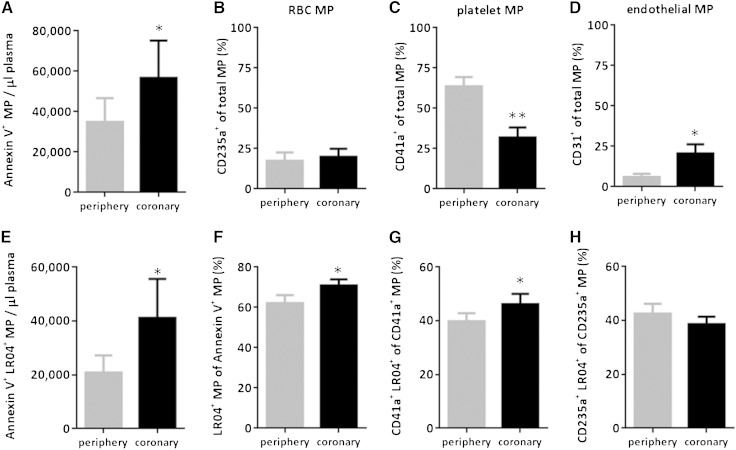
MDA-carrying MPs are increased at the culprit lesion site in STE-MI. A: Total numbers of MPs were quantified by flow cytometry in plasma isolated from peripheral (femoral artery) and coronary blood samples of patients with STE-MI. B–D: Cellular origin: Isolated MPs were stained with anti-CD41a (platelet), anti-CD235a (RBCs), and anti-CD31 (ECs) to identify their cellular origin and are presented as percentages of positive MPs of total MPs. E–H: MDA epitopes: Isolated MPs were stained with annexin V, anti-CD41a, or anti-CD235a and LR04, and analyzed by flow cytometry. Data show total numbers of annexin V^+^ LR04^+^ MP per µl plasma (E) and percentages of annexin V^+^ LR04^+^ MPs of total MPs (F), as well as the percentages of LR04^+^ MPs of either CD41a^+^ platelet (G) or CD235a^+^ RBC (H) MPs. All results are presented as mean ± SEM of 13–14 STE-MI patients; * *P* < 0.05 and ** *P* < 0.01 (paired *t*-test).

Several studies have reported that patients with ACS have increased levels of circulating MPs, which could propagate the inflammatory responses of affected arteries. Therefore, we tested whether MPs isolated from the peripheral and coronary circulations of patients with STE-MI also carry MDA epitopes. Blood was obtained from the femoral artery sheath and from the site of occlusion of the affected coronary arteries of the same patients during pPCI (supplementary Table 1). MP levels were significantly higher in the affected coronary arteries compared with the peripheral site of the same patient (**Fig. 4A**). In comparing the source of isolated MPs from the peripheral (88 ± 2% annexin V^+^ particles) and coronary circulation (81 ± 4% annexin V^+^ particles), the relative contribution of RBC-derived MPs was similar between the two vascular sites (Fig. 4B). In contrast, platelet-derived MPs were decreased (Fig. 4C), while EC-derived MPs increased in the coronary compared with the peripheral blood (Fig. 4D). Moreover, the frequency of monocyte/macrophage-derived MPs identified by expression of CD14 was very low and not different between the two sites (periphery: 1.02% ± 0.3 of total MPs vs. coronary: 1.15% ± 0.3 of total MPs). Similar data were obtained after normalization for annexin V^+^ events (data not shown). Importantly, characterization of MPs from both sites demonstrated not only higher numbers (Fig. 4E) but also a higher percentage of MDA-carrying MPs (Fig. 4F) in the coronary circulation, despite similar levels of anti-MDA-LDL IgM at both sites (supplementary Fig. 7). Moreover, the increased frequency of MDA-carrying MPs was specifically found in platelet-derived MPs (Fig. 4G), but not RBC-derived MPs (Fig. 4H). Thus, the total number and frequency of MDA-carrying MPs are elevated in the coronary circulation of STE-MI patients.

## DISCUSSION

In this study, we identified a subset of circulating MPs that carry OSEs and are increased at the site of coronary occlusion of patients with STE-MI. OSE^+^ MPs are recognized by OxLDL-specific IgM antibodies, which have the capacity to decrease proinflammatory effects of MPs.

There is substantial evidence from both epidemiological and experimental studies demonstrating that humoral immunity plays an important role in CVD (2). IgM antibodies in particular exhibit atheroprotective properties, as mice deficient in secreted IgM develop accelerated atherosclerosis (10). In line with this, low plasma IgM antibodies to MDA-LDL and Cu-OxLDL are associated with increased carotid intima media thickness and an increased risk of developing a >50% diameter coronary stenosis in humans (3, 4). Moreover, high levels of OSE-specific IgM antibodies have been shown to predict a lower risk of cardiovascular events (5, 6). Specifically, a recent analysis of the Bruneck study, which prospectively followed 765 individuals for 15 years, demonstrated that high IgM titers to Cu-OxLDL and MDA-LDL were associated with a significantly lower risk of adverse cardiovascular events (7). Our findings that MPs carry OSEs and are recognized by the same antibodies that bind Cu-OxLDL and MDA-LDL suggest a novel atheroprotective mechanism for OxLDL-specific IgM.

Patients with ACS were found to have increased circulating MPs of EC origin (21). Moreover, in a recent study of 45 AMI patients, the number of MPs in the blood collected distal to the coronary lesions with an aspiration catheter of the culprit coronary artery was also found to be increased before pPCI compared with MPs in the blood collected from the femoral artery (36). We now report that STE-MI patients have increased numbers of EC-derived MPs at the coronary occlusion site, suggesting that MPs are generated most likely from injured and dying cells at this site. In turn, these MPs may contribute to proinflammatory and procoagulatory responses during atherothrombosis. We have recently demonstrated by immunological and mass spectroscopy techniques the presence of OSEs, including a variety of OxGP as well as MDA-type epitopes, on embolized material from vulnerable plaques trapped on distal protection devices during PCI procedures (37). MDA-modified proteins induce the secretion of proinflammatory cytokines, such as IL-8, in monocytes and macrophages (16, 38). Because MPs also carry MDA epitopes, they may propagate inflammation locally by triggering inflammatory responses via the same mechanisms. In turn, these can be inhibited by MDA/MAA-specific IgM antibodies. Additional biological activities may also be mediated by other OSEs present on MPs, including PC of OxGP, which we found to be nearly exclusively present on MDA-carrying MPs. Notably, PC epitopes have been found to mediate EC activation when present on apoptotic cells or blebs (15, 31). It has been previously shown that bioactive lipid-bearing platelet-derived MPs can activate platelets and ECs (25). One may hypothesize that MDA-modified lipids or proteins on the surface of MPs contribute to these effects. Interestingly, activation of platelets has also been shown to generate MDA and induce MDA-modifications in LDL (39, 40). Whether OSE-specific IgM could also interfere with the procoagulatory potential of MPs and subsequent thrombus formation remains to be shown.

In conclusion, a subset of circulating MPs carry OSEs that are recognized by IgM NAbs, which in turn may dampen their proinflammatory properties, and may also enhance their clearance. Understanding the functional properties of the interaction between MPs and IgM antibodies may help to identify novel diagnostic and therapeutic approaches for CVD.

## Supplementary Material

Supplemental Data
