# Supplementary material for: Circulating microparticles carry oxidation-specific epitopes and are recognized by natural IgM antibodies
Source: J Lipid Res. 2015 Feb;56(2):440–8. doi: 10.1194/jlr.P054569 (PMC4306697; doi:10.1194/jlr.P054569)
Supplement: Supplemental Data [file supp_56_2_440__index.html]

Circulating microparticles carry oxidation-specific epitopes and are recognized by natural IgM antibodies — Circulating microparticles carry oxidation-specific epitopes and are recognized by natural IgM antibodies — Supplemental Data 

# Circulating microparticles carry oxidation-specific epitopes and are recognized by natural IgM antibodies

## Supplemental Data

**Files in this Data Supplement:**

- supplemental data - Supplemental data
