# Supplementary material for: Circulating microparticles carry oxidation-specific epitopes and are recognized by natural IgM antibodies
Source: J Lipid Res. 2015 Feb;56(2):440–8. doi: 10.1194/jlr.P054569 (PMC4306697; doi:10.1194/jlr.P054569)

SUPPLEMENTAL MATERIAL

Supplemental Table 1

**Number of patients**		**14**
**Age, years**		54.9 ± 10.0
**Female gender, n (%)**		6 (42.9)
**Diabetes, n (%)**		2 (14.3)
**History of hypertension, n (%)**		5 (35.7)
**Ever smoker, n (%)**		10 (71.4)
**Family history of CAD, n (%)**		6 (42.9)
**BMI > 25kg/m², n (%)**		11 (78.6)
**Troponin T max, ng/ml**		3.9± 3.2
**Culprit vessel, n (%)**	LAD	4 (28.6)
	CX	3 (21.4)
	RCA	7 (50)
**CAD, n (%)**	1VD	8 (57.1)
	2VD	6 (42.9)
	3VD	0 (0)
**CPKmax, U/l**		2076.4 ± 1947.8
**CRP, mg/dl**		0.7 ± 0.8
**Creatinine, mg/dl**		1.1 ± 0.3
**Total Cholesterol, mg/dl**		220.0 ± 60.2
**LDL, mg/dl**		136.3 ± 59.7
**HDL, mg/dl**		39.7 ± 5.8
**Triglycerides, mg/dl**		185.9 ± 188.5

Supplemental Table 1. STE-MI patient characteristics. Data are presented as mean ± SD. CPKmax, CRP, Creatinine, Cholesterol, LDL, HDL and Triglycerides were measured one day after pPCI. CAD, coronary artery disease; BMI, body mass index; LAD, left anterior descending artery; CX, left circumflex artery; RCA, right coronary artery; VD, vessel disease; CPKmax, peak value of serum creatine phosphokinase; CRP, C-reactive protein; LDL, low-density lipoprotein cholesterol; HDL, high-density lipoprotein cholesterol.

Supplemental Figure Legends:

Supplemental Figure 1. Flow-cytometric gating strategy for MP definition. The MP size gate was defined using monodisperse polystyrene reference beads with diameter of 1 µm and data acquired from buffer only to exclude noise. MP were stained with Annexin V and analyzed by flow cytometry. Representative flow cytometry plots are shown.

Supplemental Figure 2. Binding properties of monoclonal IgM antibodies. The IgM NAb T15/E06 is specific for PC, whereas LR04, NA17, and E014 are directed against MDA-type epitopes. Binding of **(A)** T15/E06, **(B)** LR04, **(C)** NA17 and **(D)** E014 to either MDA-BSA or PC-BSA or native BSA was assessed by chemiluminescent ELISA. MDA-BSA contains different MDA-type epitopes including, advanced MAA-epitopes. Data are expressed as RLU/100ms.

Supplemental Figure 3. A subset of circulating MP carries OSE.

Circulating plasma MP of 18 healthy volunteers were stained with biotinylated T15/E06 (specific for PC) and LR04 (specific for MDA/MAA) as well as an isotype control antibody and analyzed by flow cytometry. Shown are the percentages of MP with positive staining for each antibody. Bonferroni's multiple comparison test; (# $P<0.001$compared to isotype control; (§ $P<0.001$; and # $P< 0.0001$ compared to T15/E06). Circles depict MP from individual donors.

Supplemental Figure 4. PC epitopes are nearly exclusively present on MP that carry MDA epitopes.

Circulating plasma MP of healthy volunteers (n=3) were sequentially stained with T15/E06 (specific for PC), anti-mouse IgM FITC (II/41), LR04 (specific for MDA/MAA), anti-mouse IgM APC (II/41) and Annexin V and analyzed by flow cytometry. Data show dot plots of one representative experiment out of three (A) and the percentages of Annexin V^+ MP with positive staining for either LR04 or T15/E06 only or for both antibodies (B).

Supplemental Figure 5. The presence of OSE on MP is not due to ex vivo oxidation. Circulating MP were isolated by sequential ultracentrifugation from plasma treated with BHT or left untreated. Subsequently, binding of LR04 and T15/E06 to MP was quantified by flow cytometry. Data show contour plots of a representative experiment.

Supplemental Figure 6. A subset of in vitro generated platelet-derived MP carry MDA epitopes. Platelet-derived MP were isolated from ionomycin-treated platelets, and binding of LR04 to MP and parental platelets was evaluated by flow cytometry. Shown are representative flow cytometry plots of **(A)** MP and **(B)** platelets stained with either a control antibody or LR04 as indicated. In contrast to parental cells, platelet-derived MP carry MDA epitopes.

Supplemental Figure 7. Similar IgM antibody titers specific for MDA-LDL in coronary and peripheral plasma of STE-MI patients. (A) Determination of IgM titers to MDA-LDL in diluted plasma isolated from peripheral and coronary blood of STE-MI patients by ELISA. Data are mean ± SEM of 14 AMI patients and expressed as RLU/100ms. **(B)** Correlation of IgM titers to MDA-LDL between peripheral and coronary circulation. Circles represent results of individual patients and correlation analysis was performed with Pearson's test; $P<0.001$.

Supplemental Figure 1. Flow-cytometric gating strategy for MP definition

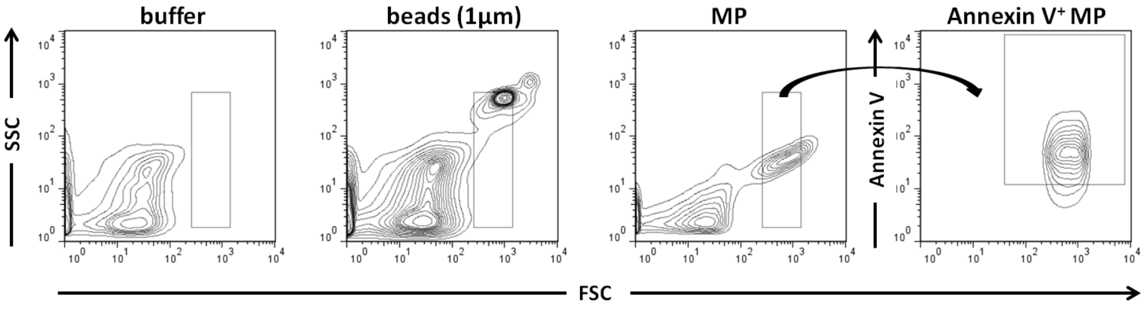

Supplemental Figure 2. Binding properties of monoclonal IgM antibodies

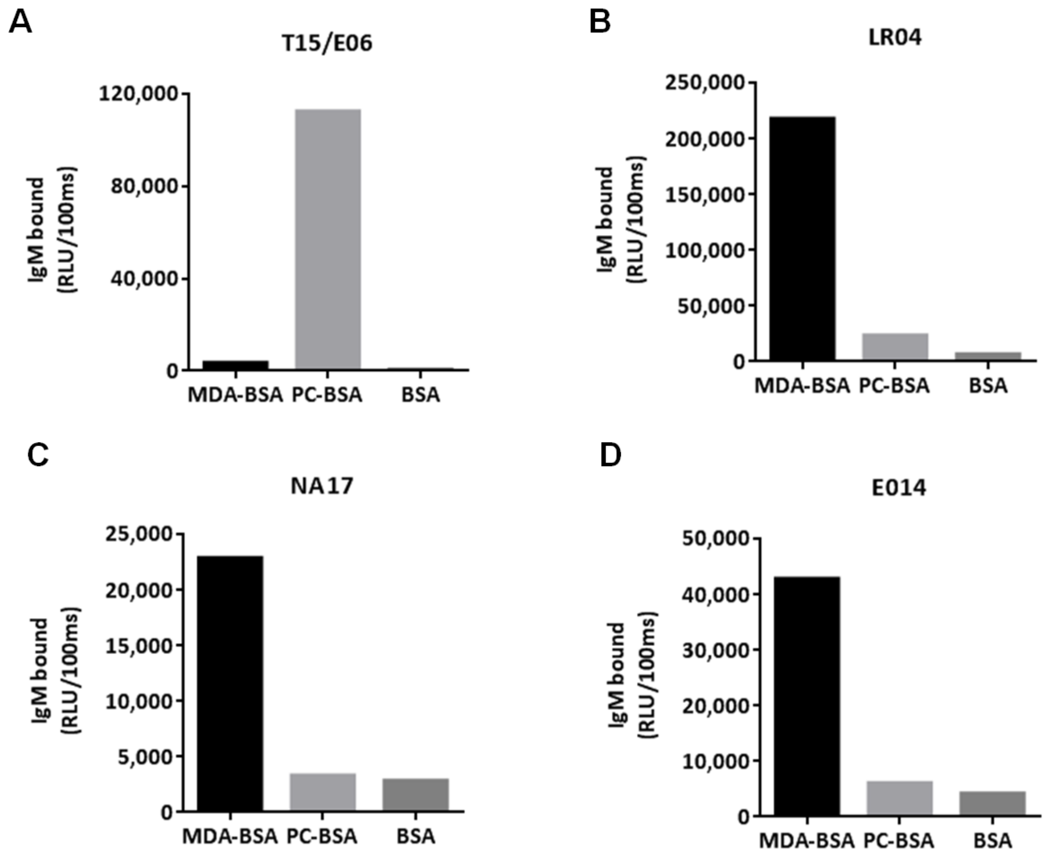

Supplemental Figure 3. A subset of circulating MP carries OSE

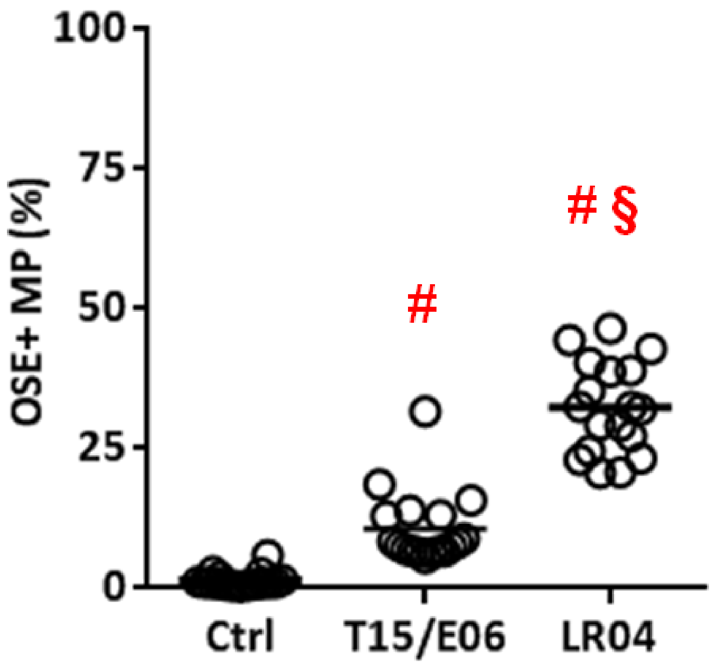

Supplemental Figure 4. PC epitopes are nearly exclusively present on MP that carry MDA epitopes

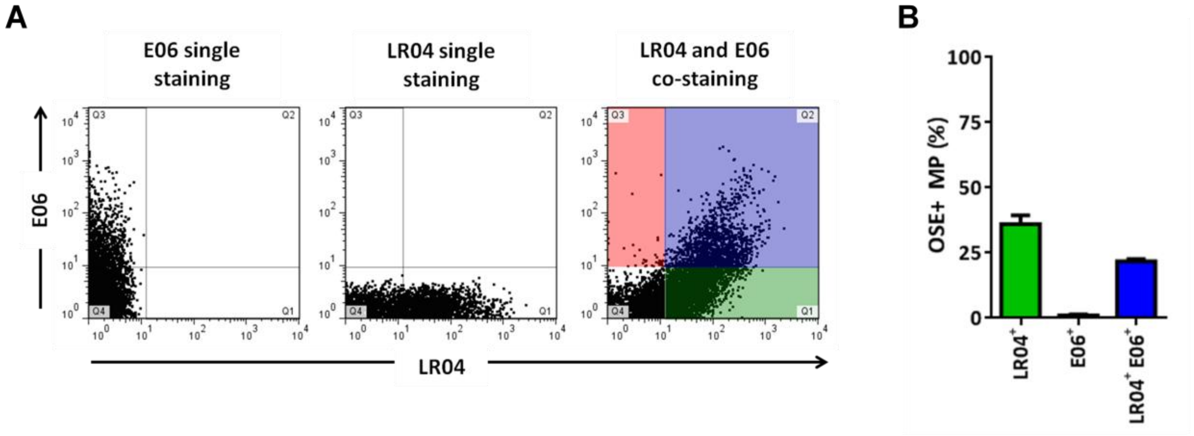

Supplemental Figure 5. The presence of OSE on MP is not due to ex vivo oxidation

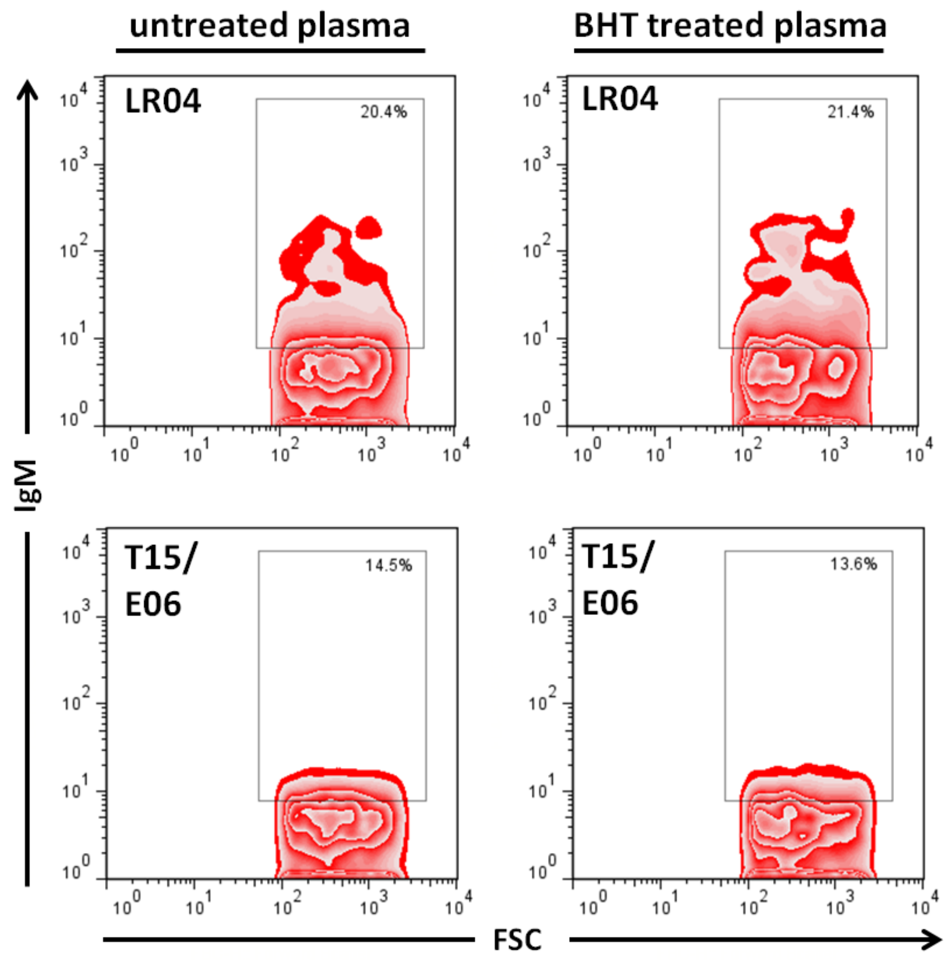

Supplemental Figure 6. A subset of in vitro generated platelet-derived MP carry MDA epitopes

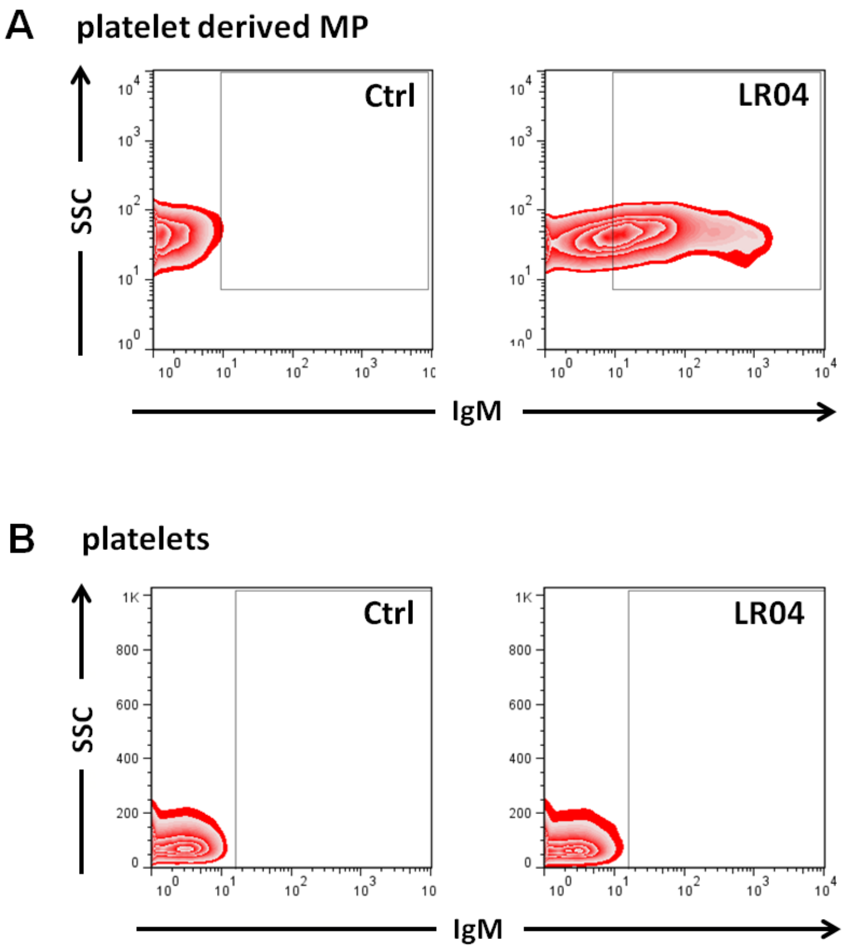

Supplemental Figure 7. Similar IgM antibody titers specific for MDA-LDL in coronary and peripheral plasma of STE-MI patients

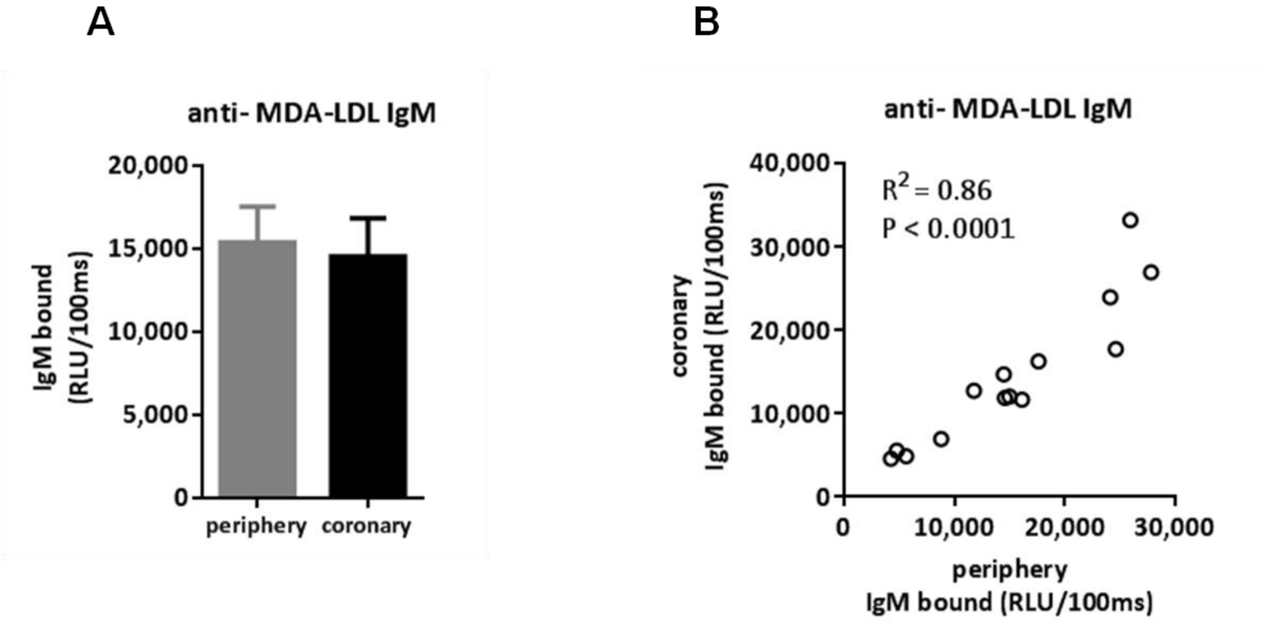

Supplement: Supplemental Data [file supp_P054569_jlr.P054569-1.pdf]
